# Cysteine carboxyethylation: a novel post-translational modification associated with autoimmune arthritis

**DOI:** 10.1038/s41392-023-01532-2

**Published:** 2023-07-19

**Authors:** Alessandro Didonna

**Affiliations:** Department of Anatomy and Cell Biology, Brody School of Medicine, East Carolina University, Greenville, NC 27834 USA

**Keywords:** Immunological disorders, Rheumatic diseases

In a study recently published in *Science*, Zhai et al. reported that protein carboxyethylation results in the formation of neoantigens with the ability to trigger autoimmune arthritis in the context of ankylosing spondylitis (AS) pathology.^1^ This work defines a novel molecular mechanism underlying immune tolerance breakdown, which could serve as a target for innovative therapeutic strategies against AS and other autoimmune disorders.

The authors performed a mass-spectrometry screening of all post-translational modifications (PTMs) in the proteome of peripheral blood mononuclear cells (PBMCs) isolated from AS patients and healthy controls, and a significant increase in integrin αIIb carboxyethylated at cysteine 96 (ITGA2B-ceC96) was detected in the AS cohort. Comparative analysis between ITGA2B and ITGA2B-ceC96 interactomes identified cystathionine β-synthase (CBS) as the enzyme catalyzing cysteine 2-carboxyethylation, while 3-hydroxypropionate (3-HPA) was characterized as the donor of the carboxyethyl group. The subsequent development of an antibody specific for ITGA2B-ceC96 confirmed the presence of the modified protein in vivo.

Fueled by these observations, Zhai and colleagues explored the consequences of this novel PTM on ITGA2B structure and function. Cycloheximide chase assays demonstrated that ITGA2B-ceC96 displays reduced stability without affecting cell viability. The disruption of a disulfide bridge between C87-C96 residues seems to be responsible for the shorter half-life of the modified protein as suggested by the evidence that mutations blocking the formation of this bond similarly increase ITGA2B clearance. The lysosomal degradation pathway likely mediates this molecular phenotype since the lysosome inhibitor bafilomycin A1 was shown to stabilize ITGA2B-ceC96.

Next, Zhai and colleagues evaluated the immunogenic potential of the peptides originated from enhanced ITGA2B-ceC96 degradation. The authors tested plasma samples from AS and healthy donors for the presence of ITGA2B-ceC96-specific antibodies. Higher levels of autoantibodies were found in AS patients compared to controls, thus supporting the notion that modified ITGA2B peptides can function as neoantigens and elicit an autoimmune response. Consistently, dendritic cells pulsed with ITGA2B-ceC96(84-110) peptides are able to promote the proliferation and activation of CD4^+^ T cells isolated from autoantibody-positive AS donors. Human leukocyte antigen (HLA) typing of these AS patients showed an association between autoantibody titers and HLA-DRB1*04 carriage, possibly implicating HLA-DR4 heterodimers in the presentation of ITGA2B-ceC96 peptides to T lymphocytes. Such possibility was experimentally verified in binding experiments employing HLA-DRB1*04–ceC96 tetramers and Jurkat cells expressing T cell receptor (TCR) molecules cloned from AS patients. Likewise, peptide competition assays and in silico docking simulations highlighted specific interactions between ITGA2B peptides spanning the ceC96 residue and the antigen-binding pocket of the HLA-DRB1*04/HLA-DRA*01 heterodimer.

In a last set of experiments, Zhai and colleagues sought to dissect the contribution of ITGA2B carboxyethylation to AS pathogenesis in vivo. Remarkably, HLA-DR4 transgenic mice immunized with ITGA2B-ceC96(84-110) peptide and treated with 3-HPA developed extensive vertebral bone erosion in the lumbar spine and colitis—a common extraskeletal manifestation of AS pathology. Colon inflammation was associated with the presence of peptide-specific T cells and immunoglobulins as well as with granulocytes and neutrophils in the lamina propria of colonic mucosa.

**Fig. 1:**
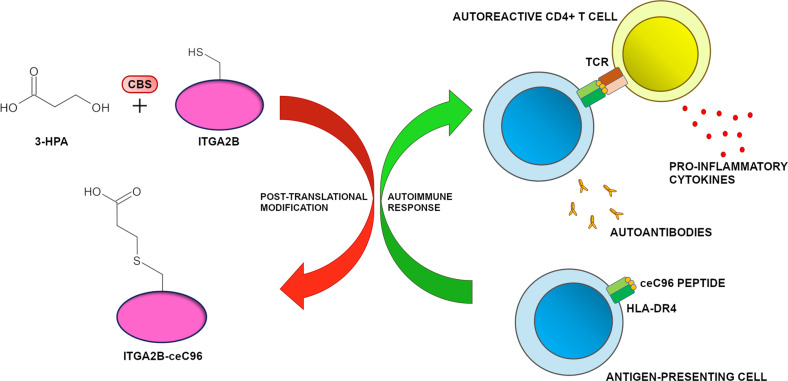
Proposed model for ankylosing spondylitis (AS) pathogenesis. In specific metabolic conditions associated with increased levels of 3-hydroxypropionate (3-HPA), the enzyme cystathionine β-synthase (CBS) mediates the post-translational modification of cysteines via the addition of carboxyethyl groups. In the peripheral immune system, carboxyethylation of C96 residue in integrin αIIb (ITGA2B-ceC96) results in higher degradation of the modified protein with the consequent formation of immunogenic carboxyethylated peptides. Such peptides are presented by HLA-DR4 expressing professional antigen-presenting cells (APCs) to autoreactive CD4^+^ T cells which in turn start an aberrant immune response against AS target tissues. Cell-mediated autoimmunity is also accompanied by the secretion of autoantibodies against ITGA2B-ceC96, which further exacerbates AS pathology. All chemical structures were drawn using ChemSketch software

In line with multiple genetic and epidemiological observations,^2,3^ the work by Zhai and colleagues corroborates the autoimmune etiology of AS and pinpoints aberrant PTM patterns as a critical mechanism for pathogenic neoantigen generation. PTMs typically take place in peripheral target tissues; hence, high-affinity T cells recognizing the modified proteins are not subjected to deletion through negative selection in the thymus. Several PTMs have been previously linked to autoimmunity.^4^ Citrullinated autoantigens are responsible for initiating inflammation in synovial tissues of rheumatoid arthritis (RA) patients. Deamidated peptides are instead the targets of CD4^+^ T cells in type 1 diabetes (T1D) and coeliac disease. The authors add to this picture a working model for AS pathogenesis in which metabolic changes cause an increase in 3-HPA levels which in turn drives autoimmunity via cysteine carboxyethylation of specific target proteins (Fig. 1). Such a scenario is in agreement with the evidence that AS patients display elevated 3-HPA blood levels. Also, gut microbes synthesizing 3-HPA, such as the bacteria of the genus *Klebsiella*, are known to exacerbate AS manifestations.^5^ In this light, the metabolic pathway responsible for 3-HPA production may represent a novel druggable target for possible therapeutic intervention to tackle AS. For instance, small molecules or biologics blocking CBS catalytic activity could be employed to reduce the formation of pathogenic neoepitopes in the immune system of people at risk. Alternatively, approaches aimed to correct the gut dysbiosis affecting AS patients could be instrumental to reduce circulating 3-HPA levels. Lastly, chimeric antigen receptor (CAR) T cells could be designed to directly recognize and remove autoreactive T cells, neoantigen-presenting cells, or autoantibody-secreting plasma cells.

Intriguingly, this newly characterized cysteine PTM may represent a broad pathogenic event with implications for other autoimmune disorders. In fact, Zhai and colleagues have documented the presence of carboxyethylated ITGA2B also in PBMCs from patients with RA and systemic lupus erythematosus (SLE). In the future, it will be crucial to understand whether cysteine carboxyethylation mechanistically contributes to the etiology of these diseases or represents a mere epiphenomenon.
